# Ion-selective membranes by UV-induced cleavage of polymer chains

**DOI:** 10.1093/nsr/nwaf439

**Published:** 2025-10-15

**Authors:** Young Moo Lee

**Affiliations:** Department of Energy Engineering, Hanyang University, Republic of Korea

Creating angstrom-sized channels in polymer membranes remains one of the central challenges in molecular and ionic separations. The separation of small molecules such as gases, water and ions requires precisely defined pathways at the sub-nanometer scale [1]. While materials such as graphene, metal–organic frameworks (MOFs) and covalent organic frameworks (COFs) offer well-defined sub-nanochannels, their limited scalability hinders their industrial application. In contrast, polymer membranes are scalable and versatile, yet they often suffer from a trade-off between permeability and selectivity [2]. Conventional phase-inversion processes typically produce broad pore-size distributions lacking the uniform angstrom-level channels required for true molecular sieving.

**Figure 1.**
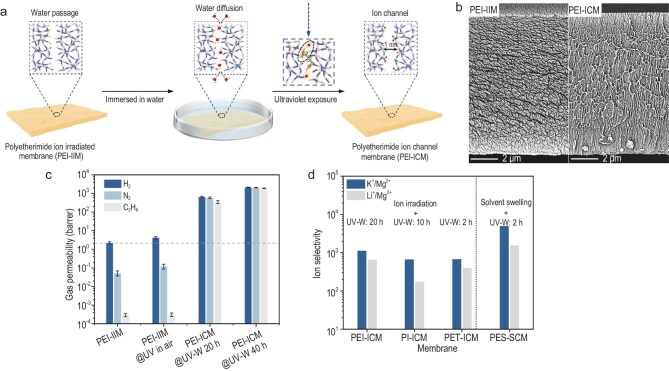
(a) Schematic illustration of the UV–W-induced process for preparing PEI-ICM. Following ion irradiation and immersion in water, PEI membranes allow water to diffuse into the latent ion tracks; upon UV exposure, reactive species generated in water cleave polymer chains, forming angstrom-sized ion transport channels. (b) Cross-sectional morphology of PEI-IIM and PEI-ICM. (c) Gas permeabilities of membranes prepared with different conditions measured at 30°C and 1 bar. (d) K^+^/Mg^2+^ and Li^+^/Mg^2+^ selectivity of polymer membranes fabricated through the ion irradiation @UV–W process and solvent swelling @UV–W process. Adapted from Cheng *et al*. [3].

In a recent study, Cheng *et al.* [3] introduced a unique approach to engineer angstrom-sized channels in polymers through a UV–water-induced (UV–W) scissoring process in ion-irradiated membranes (Fig. 1a). This method differs fundamentally from traditional ion-track etching techniques, which rely on heavy ions (e.g. Ta, Bi, Au) and post-etching to generate large, nanometer-scale pores [4]. Instead, the UV–W process leverages water as a reactive medium under UV illumination to generate highly oxidative hydroxyl radicals (•OH), which locally cleave polymer chains and create ultrafine channels.

The authors employed polyetherimide ion-irradiated membranes (PEI-IIMs) as precursors. When immersed in water and exposed to UV light, photolysis of water molecules generates hydroxyl radicals that attack polymer chains within the irradiated tracks, leading to chain scission and the formation of new oxygen-containing functional groups. The resulting PEI ion-channel membrane (PEI-ICM) exhibits angstrom-sized, hydrophilic channels that enable selective ion transport (Fig. 1b). Remarkably, the channel size can be tuned by controlling UV exposure time, enabling precise modulation of ion selectivity.

Characterization using positron annihilation lifetime spectroscopy (PALS) confirmed the formation of tunable angstrom-scale free-volume channels. These membranes achieved outstanding ion selectivity, with Li⁺/Mg²⁺ selectivity exceeding 1000 and Li⁺ permeation rates up to 0.3 mol m⁻² h⁻¹, surpassing existing polymer membranes by orders of magnitude (Fig. 1c). By contrast, conventional ion-track etched membranes with 20 nm pores exhibited no ion selectivity.

This UV–W strategy provides a controllable and scalable method for creating sub-nanometer transport pathways in glassy polymers such as PEI, poly(ethylene terephthalate) (PET) and polysulfone (PES) (Fig. 1d). Beyond ion separation, such membranes could have far-reaching implications for energy and environmental applications, including lithium recovery, desalination and selective molecular separations.

This work demonstrates that polymer chain scission, traditionally regarded as a degradation pathway, can be transformed into a precise nanofabrication tool—enabling a new class of ion-selective membranes with molecular-level precision.
